# Perceptions and Experiences of Intimate Partner Violence in Abidjan, Côte d'Ivoire

**DOI:** 10.1371/journal.pone.0157348

**Published:** 2016-06-16

**Authors:** Sara J. Shuman, Kathryn L. Falb, Lauren F. Cardoso, Heather Cole, Denise Kpebo, Jhumka Gupta

**Affiliations:** 1 School of Nursing and Health Sciences, La Salle University, Philadelphia, PA, United States of America; 2 International Rescue Committee, New York, NY, United States of America; 3 School of Social Policy and Practice, University of Pennsylvania, Philadelphia, PA, United States of America; 4 Innovations for Poverty Action, Abidjan, Côte d’Ivoire; 5 Department of Global and Community Health, College of Health and Human Services, George Mason University, Fairfax, VA, United States of America; Harvard Medical School, UNITED STATES

## Abstract

**Background:**

Men and women’s perceptions of intimate partner violence (IPV) within crisis-affected populations are not well understood. This mixed-methods study examined the frequency of IPV against women in urban Cote d’Ivoire, and qualitatively explored how men and women perceive the impact of various forms of IPV on health, everyday activities, and feelings of shame.

**Methods:**

A survey was administered to Ivorian women (N = 80) to measure the frequency of IPV, and ten focus group discussions were conducted with women (n = 46) and men (n = 45) to explore perceptions of different forms of IPV, including its impacts on disruptions to health, everyday activities, and experiences of shame.

**Results:**

Half of all surveyed women (53.6%) reported past year exposure to physical, sexual, or emotional IPV. Of the multiple types of violence, emotional IPV was most common (46.4%), followed by sexual IPV (21.7%) and physical IPV (17.4%). Focus group participants identified additional forms of violence including economic IPV and community discrimination. Lack of financial resources and unemployment were common problems among crisis-affected women and were described as an underlying source of IPV. Both women and men reported that shame and stigma play a large role in how women experience the repercussions of IPV, regardless of the form of violence, with public episodes of IPV almost always seen as more detrimental than private episodes of IPV.

**Conclusions:**

These results underscore the need for increased social support mechanisms for women to reduce the shame, stigma, and isolation associated with their experiences. The creation of safe and supportive spaces for women to talk about and challenge social norms may be an important first step in reducing community shaming and the secrecy that often surrounds IPV. Safe spaces along with broader societal outreach, including challenging men’s social positions and creating opportunities for increasing economic resources can, in turn potentially decrease the frequency of IPV and its deleterious impacts on a woman’s well-being

## Introduction

Intimate partner violence (IPV) against women in crisis and post-crisis populations is an important area of public health research still in its nascent stage. Although data are limited, available studies indicate that individual exposure to armed conflict or crises may exacerbate IPV, with several studies finding a quantitatively measured or qualitatively perceived positive association between exposure to political violence and women’s vulnerability to experiencing IPV [1–6]. For example, a cross-sectional study in Palestine found that married women with partners exposed to political violence were significantly more likely to experience physical and sexual IPV compared to women whose partners were not exposed to political violence [2]. In a qualitative study of the causes of IPV in post-conflict Sierra Leone and Liberia, some women believed that men participating in or witnessing violence during conflict was a possible risk factor for IPV perpetration [7]. Prior research posits that increases in post-crisis IPV are likely the result of a complex interaction of factors, including but not limited to increased community violence, shifts in gender roles, economic stress, and displacement [8].

While studies of the frequency and risk factors of IPV in crisis and post-crisis settings have started to emerge, there is little information about how crisis affected women and men conceptualize IPV, and how such experiences impact their day to day lives. Such understanding is needed to provide important insights into culturally appropriate and tailored IPV prevention and response programming.

Côte d’Ivoire, having emerged from a period of crisis and instability in the mid-2000’s, recently experienced a new wave of political violence and displacement after a contested 2010 presidential election. Intense fighting ensued following the election and members of both political parties committed human rights abuses and crimes including murder, rape, and political persecution. In a span of six months, at least 3,000 civilians were reported killed, thousands were internally displaced, and many more were injured or witnessed violence [9]. In the majority of cases, these internally displaced people (IDPs) moved to Abidjan and lived with other families [10]. Previous research with the same population as the present study found that unemployment, food insecurity, housing instability and a lack of social networks were common concerns of IDPs [11]. Prior community-based research in rural Côte d’Ivoire has also documented a high prevalence of IPV among rural women, with 60% of surveyed women reporting experiencing lifetime IPV, and past-year estimates ranging from 20–23% [12]. The current study makes an important contribution by measuring the frequency and describing the experiences of IPV in an urban Ivorian population that experienced armed conflict and widespread election-related violence. The perspectives of both women and men were solicited, including descriptions of how multiple forms of IPV are experienced by women, particularly as related to health, everyday activities, and shame. This study adds to a small but growing body of work about IPV in post-crisis situations.

## Materials and Methods

This study was a sequential mixed methods study. The first step was to administer a cross-sectional survey with urban crisis affected women. Next, focus group discussions were conducted with women and men to increase understanding of the experiences women described in the survey. All data were collected between 2012 and 2014.

### Quantitative Survey

In July of 2012, eighty women were systematically sampled from households in the Abobo neighborhood of Abidjan, Côte d’Ivoire to complete a one-time survey about economic autonomy, decision-making, violence, reproductive health, and the post-election crisis. This survey was undertaken as part of piloting activities for the endline survey of a randomized controlled trial occurring in rural villages [12]. The Abobo neighborhood was selected for the survey due to the concentration of crisis-related violence, dense population, low socio-economic status, and ethnic diversity including IDPs. Four of Abobo’s 10 quartiers were randomly selected to participate in the survey. The research team first met with each quartier’s leader to obtain permission to conduct the survey and households were systematically sampled by a locally trained, Ivorian French-speaking enumerator. Eligible women (aged 18 or older) were invited to participate. Of the one hundred women approached, 17 did not consent to participate due to lack of time (n = 14), partners’ disapproval (n = 2), or another reason (n = 1); yielding a final sample of 80 participants.

Language-matched, trained female research assistants administered paper-based surveys concerning women’s experiences with different forms of IPV and socio-demographic characteristics in Abobo in July 2012. The survey to assess gender-based violence in Côte d’Ivoire was adapted from three previous studies: the WHO Multi-country Study on Women’s Health and Domestic Violence, the London School of Hygiene and Tropical medicine violence and health among women asylum seekers, and an HIV and IPV prevention trial in Uganda [13–16]. The instrument also contained additional items (e.g. household decision making, exposure to conflict) to gauge other facets of women’s lives. Descriptive statistics were used to examine the prevalence of IPV and participants’ socio-demographic characteristics.

### Focus Group Discussions

Ten focus groups were conducted in May and June 2014 (N = 46 women; N = 45 men). Focus group participants were identified by community leaders in the Abobo neighborhood in collaboration with the International Rescue Committee (IRC) and its community partners. The IRC is an international humanitarian organization with an established history in Côte d’Ivoire. Community members aged 18+ were invited to a series of meetings where IRC presented the purpose of the study. These meetings resulted in the formation of six focus groups: (1) 10 Non-IDP women; (2) 10 Non-IDP men; (3) 10 IDP women; (4) 10 IDP men; (5) 10 IDP women; (6) 9 IDP men. Eligibility criteria for all focus groups included being at least 18 years old, French speaking, and having a partner interested in joining the focus groups discussions. Four additional gender-stratified focus groups were formed with women participating in Urban Savings and Loan Associations (USLAs), an IRC group savings program in Adjame and Treichville, neighborhoods sharing socioeconomic and population density characteristics with Abobo. Focus groups seven through ten were composed of: (7) 6 USLA women; (8) 7 USLA men; (9) 10 USLA women; (10) 9 USLA men. USLA groups were facilitated separately to see if program participants had a different perspective about IPV contributing factors and solutions to reduce IPV in urban settings compared to those who did not participate.

Two Ivorian facilitators led each group, with two note-takers present. The interview guide included semi-structured questions, free listing exercises, and pair-wise ranking activities to explore the following: a) how women, families, and communities were impacted by election-related violence, and b) investigate how women and men understand IPV in relation to shame, health, and its impact on their everyday life. Free listing produces inventories (lists) that offer insight into the salience of items among respondents [17]. During pair-wise ranking, participants were asked to compare two different forms IPV (e.g. “Of physical IPV and economic IPV, which is the more important of the two?”) and discuss each in terms of shame, health, and impact on their everyday activities. This methodology was used to stimulate conversations about specific forms of IPV. Facilitators guided interview participants through the free listing and pair-wise ranking processes. The goal of the pair wise ranking was not necessarily to reach consensus among participants, but rather a methodological tool to explore perceptions and thought-processes regarding the impacts of different forms of IPV. Focus groups lasted approximately two and a half hours.

Focus group discussions were recorded using digital audio recorders. Each recording was transcribed verbatim in French by a native Ivorian French speaker and then translated to English by professional translators. A trained bilingual Ivorian researcher compared the English translations to the French transcriptions to confirm accuracy and clarify any terms specific to the region. Atlas.ti® software was used to organize and code the transcripts and a grounded theory approach was used in data analysis. Grounded theory emphasizes an inductive, open-ended approach towards data analysis, encouraging the elimination of preconceived ideas, shifting to the generation of a list of emergent themes [18]. Each interview transcript was coded in English by two researchers (L.G., S.S.) with descriptive statements created to reflect emerging themes. Any discrepancies in coding were discussed with the principal investigators (J.G., K.F). Codes were then organized into themes across the interviews through an iterative process and reviewed by the principal investigators and coding team. During data collection for each focus group, a note taker carefully recorded free listing and pair-wise ranking results. Free listing responses were recorded on paper and ranking results were entered into a matrix.

All survey and focus group participants provided signed informed consent prior to participating. This study received ethical approval from the Institutional Review Board at Yale University, George Mason University, and was reviewed by the Ivorian National Ethical Review Board.

## Results

### Participant characteristics

A total of 80 women completed the survey, of which 86.25% (n = 69) reported having a male intimate partner within the last year. Approximately 9% (n = 6/69) of partnered women were in polygamous marriages. IDP status was not collected as part of the quantitative survey, as advised by the IRC, in order to minimize feelings of stigma among IDPs, as quantitative data collection was conducted close to the time of election violence.

Similar to the survey sample, focus group participants were largely partnered (95.7%, n = 44) and 42.9% were IDPs (n = 20). Seventy-six percent of focus group respondents were employed (n = 35) and three were students.

### Frequency of IPV: Quantitative Results

Half (53.6%) of women (partnered and non-partnered) in the survey sample reported experiencing emotional, physical, and/or sexual IPV during the past year. Of the multiple types of violence, emotional IPV was most common (46.4%), followed by sexual IPV (21.7%), and physical IPV (17.4%). Nearly one in three (29.0%) women reported experiencing past year physical and/or sexual IPV. Among partnered women, being pushed, shoved, kicked or dragged (14.5%) and being slapped, having something thrown, or being hit with something (13.0%) were most common forms of physical IPV. Being forced to have sex as a result of threats or intimidation (15.9%) and being physically forced to have sex (11.6%) were the most common types of sexual IPV, and feeling frightened or humiliated (36.2%) was the most common form of emotional IPV among partnered women.

### Perceptions of IPV: Qualitative Results

During the focus groups, women and men first created lists of problems and discrimination experiences faced by crisis-affected women. Afterwards, they created matrices and discussed how different forms of IPV were related to feelings of shame, overall health, and ability to perform everyday activities. Results from the free-listing activity and pair-wise ranking discussion are presented below. During both activities, participants were provided with time to discuss, explain, and provide examples for their choices. Supporting quotes from these discussions are presented in the following sections. Although considerable overlap existed between women and men, gender differences are highlighted when relevant.

### Free-Listing Results

The free-listing prompted focus group participants to create lists of 1) general problems and 2) types of IPV, violence, and discrimination experienced by crisis-affected women in Côte d’Ivoire. Results by gender are presented in Table 1.

**Table 1:** Free Listing Results from Focus Group Interviews by Gender (n = 91).

**Question 1: What types of problems do women who have been affected by election-related violence face in your community?**			
	Total	Women	Men
	n = 91 (%)	n = 46 (%)	n = 45 (%)
Financial Problems	16 (20.8%)	10 (21.7%)	6 (13.3%)
Unemployment/lost business	11 (12.1%)	7 (15.2%)	4 (8.9%)
Mental Health Repercussions/Trauma	11 (12.1%)	5 (10.9%)	6 (13.3%)
Responsibility for managing household	10 (11.0%)	7 (15.2%)	3 (6.7%)
Death of Family Member	6 (6.6%)	2 (4.3%)	4 (8.9%)
Forced Prostitution or Transactional Sex	5 (5.5%)	2 (4.3%)	3 (6.7%)
Loss of Material Goods	5 (5.5%)	4 (8.7%)	1 (2.2%)
Sexual Violence/Rape (non-IPV)	5 (5.5%)	0	5 (11.1%)
Food Insecurity	4 (4.4%)	3 (6.5%)	1 (2.2%)
Physical Health Repercussions	3 (3.3%)	2 (4.3%)	1 (2.2%)
Housing Insecurity	3 (3.3%)	3 (6.5%)	0
Abandoned by Partner/Separation/Divorce	3 (3.3%)	1 (2.2%)	2 (4.4%)
Poor Living Conditions	2 (2.2%)	1 (2.2%)	1 (2.2%)
Traumatized Children	2 (2.2%)	1 (2.2%)	1 (2.2%)
Partner is unemployed/lost business	1 (1.1%)	1 (2.2%)	0
Displacement	1 (1.1%)	0	1 (2.2%)
Physical Intimate Partner Violence (IPV)	1 (1.1%)	1 (2.2%)	0
Emotional/Financial IPV	1 (1.1%)	1 (2.2%)	0
**Question 2: What types of violence or discrimination do crisis-affected women usually experience?**			
	Total	Women	Men
	n = 91 (%)	n = 46 (%)	n = 45 (%)
Physical IPV	13 (14.3%)	7 (15.2%)	6 (13.3%)
Economic IPV	13 (14.3%)	8 (17.4%)	5 (11.1%)
Arguments with Partner	11 (12.1%)	4 (8.7%)	7 (15.6%)
Sexual IPV	10 (11.0%)	5 (10.9%)	5 (11.1%)
Emotional IPV	8 (8.8%)	6 (13.0%)	2 (4.4%)
Discrimination from Community Members	8 (8.8%)	6 (13.0%)	2 (4.4%)
Partner Controlling Behavior	4 (4.4%)	4 (8.7%)	0
Neglect by Partner	3 (3.3%)	3 (6.5%)	0
Discrimination from Family Members	3 (3.3%)	2 (4.3%)	1 (2.2%)
Discrimination in House	2 (2.2%)	2 (4.3%)	0
Infidelity	2 (2.2%)	1 (2.2%)	1 (2.2%)

The general problems faced by crisis-affected women were wide ranging, although financial concerns were the most frequently mentioned problem by both women and men (20.8% of focus group participants). Following financial problems and unemployment, women were most likely to name increased responsibility for managing the household (15.2%) and mental health problems and/or trauma (10.9%) as common problems. Men identified similar concerns for crisis-affected women, although they were more likely to identify sexual violence by a non-intimate partner as a problem for women (11.1% of men compared to 0% of women). A smaller number of women mentioned housing insecurity, partner’s unemployment, physical IPV, and emotional IPV as problems facing crisis-affected women (6.5%, 2.2%, 2.2%, and 2.2% respectively), while no men mentioned these areas.

When facilitators asked participants to describe the types of violence or discrimination that crisis-affected women usually experience, physical (14.3%), economic (14.3%), sexual (11.0%), and emotional (8.8%) IPV were mentioned in focus groups with women and men. Arguments with partners (12.1%) and discrimination experienced from community members (8.8%), often in the form of name-calling, gossiping, or social exclusion, were also cited as problems by women and men. More women than men mentioned different forms of IPV as a problem for women, except for sexual IPV, which was mentioned by approximately 11% of women and men. Having a controlling or neglectful partner and facing discrimination in the household were issues identified by women but not men.

### Pair-wise Ranking Discussion Results

In the pair-wise ranking activity, focus group participants were asked to compare and contrast different forms of IPV in relation to shame, health, and everyday activities. For example, a facilitator prompted the group by saying “Now we are going to compare types of violence and you’ll tell us the most shameful type for a woman. Between a man who beats his wife and a man who insults his wife, what will be the most shameful situation for a women?” These questions were repeated to compare each type of IPV for three domains: shame, health, and everyday activities. Results are discussed by theme below.

#### IPV and shame

Focus group participants were asked to compare each type of IPV in relation to feeling shame. In general, women considered emotional violence to be the most shameful, even when compared to physical and sexual IPV. For example, when asked to compare the shame associated with emotional IPV versus sexual IPV, one woman commented:

*I think insulting you is the most shameful*. *Although forcing you [rape] is painful as well*, *insults will happen in front of people*. *If you don’t want to have sex*, *he will do it and it will pain you…but you cannot batter him*, *you cannot do anything for that [sexual issues]…*. *But when he insults you it’s always the same thing*. *It’s as if it was stuck on you and it follows you everywhere” (Female*, *30*, *Trader)*

This quote illustrates not only that emotional IPV was perceived as very shameful to women, but also that shame was closely tied to social stigma that could be felt more depending on if the IPV was perpetrated in public or in private. There was broad consensus that IPV perpetrated in public was shameful and resulted in social stigma. Women expressed concern that community members may gossip, lose respect for, or use their knowledge of the IPV as a way to humiliate and insult women. Therefore, deciding whether something was shameful was highly dependent on who witnessed or was aware of the abuse. For example, insulting a woman in public was described as much more shameful than insulting a woman in private. This concern is highlighted in the following quote:

*“You know*, *when people do not know what happens in your household*, *they keep respecting you*. *But once they realize that you’re regularly battered by your husband*, *you lose this respect they were showing to you*. *They do no longer have any consideration for you and this is why we are so concerned about the others’ ‘eyes*.*’ It is more shameful for us when we’re being battered in front of them*.*” (Female*, *34*, *Hairdresser*, *IDP)*

Furthermore, participants resided in a city where presumably they would be more anonymous due to the larger population. However, because they were often living in close or cramped areas, they did not have any sense of privacy. Neighbors hearing, witnessing, or seeing the results of violence were commonly mentioned as concerns by women. For IDPs, the urban environment and lack of privacy was a contrast to their experiences in rural regions. For example, one female IDP raised the issue of shame by having neighbors mock her as a result of IPV. Another discussed how she felt less social support and that this is different than her experiences before moving to Abidjan.

*“Although we are in urban areas*, *here*, *we are living in common courtyards houses*. *In these kinds of houses*, *with common courtyard*, *you can’t really have a private life*. *The houses are so close to each other and the courtyard is common*, *so everybody will hear what’s happened in your household*. *If your husband batters you all the time*, *everybody will know and you’ll no longer be respected by the neighbors*, *even their children will disrespect you*. *That’s why we are so concerned about the others’ ‘eyes*.*’” (Female*, *34*, *Hairdresser*, *IDP)*

*“When a woman has some issues in her couple*, *there is nobody to help her*. *Neighbors are just there for mocking her*. *I think the lack of social cohesion may explain that there is more violence in urban couples as compared to [rural] ones*.*” (Female*, *36*, *Unemployed*, *IDP)*

There was less consensus among men regarding the shame of emotional IPV for women, with men viewing physical and economic abuse to be more or similarly shameful for women in comparison to other forms of violence. For example, physical violence as more shameful than emotional violence emerged in three of the five men’s focus groups.

*“I said earlier that when you batter your wife you put a little detail on her face as she goes out she feels ashamed because the detail is still there*, *but the shame disappears as the detail disappears eh* … *if it swells up*, *there will be shameful until it brings down*.*” (Male*, *54*, *Artist*, *Non-IDP)*

While the above quote is focused on the relationship between shame and physical violence, similar to women, the element of public shame is important. Physical violence was seen as shameful because others could detect that a woman was beaten. The shame did not appear to be a result of actually being abused or having an abusive partner, but rather as a result of evidence of the abuse that can be seen by others.

In summary, results from pair-wise ranking revealed that women considered emotional IPV, particularly in the form of insults, to be the most shameful. The same consensus was not seen among the men’s groups. However, IPV that could be identified by others, regardless of the specific form, was perceived as shameful because it resulted in social stigmatization, and in some cases, additional abuse from non-intimate partners (i.e. neighbors). Urban living circumstances, including crowded living and closely built homes, appeared to exacerbate the shame of public IPV.

#### IPV and health

Next, participants were asked to compare and describe how different forms of IPV impacted the health of crisis-affected women. Health was defined broadly and could be inclusive of both physical and mental health. Both women and men identified physical IPV followed by sexual IPV as most negatively impacting the health of women.

*“Battering is painful*. *I have six teeth because after my husband battered me*, *I lost almost all my teeth*.*” (Female*, *33*, *Trader*, *IDP)*

*“The woman might be traumatized [sexual IPV]*. *For example*, *when night falls*, *she begins to be anxious*. *She feels uneasy*. *She already worries about what will happen*. *She panics*. *It might cause several diseases*, *stress*, *ulcers*, *and diabetes… She will be wasted slowly*.*” (Female*, *33*, *Trader*, *IDP)*

Through discussion it became clear that women did not perceive the relationships between different forms of IPV and their health impacts to be distinct. For example, physical and sexual IPV were often described as simultaneously co-occurring within an episode, thus making it difficult to assess which was more harmful to health. This was not surprising as sexual abuse often involves the threat or use of physical violence [19]. Descriptions of simultaneously co-occurring IPV and related health impacts are shown below:

*“There are some men who are quite violent*. *They can beat you to have sex with them*. *There are some women who are disfigured*, *their husbands beat them*. *When you ask*, *there is not any other cause but that [sex]*. *So he will brutalize you and he will beat you…” (Female*, *30*, *Trader*, *non-IPD)*

*“There are a lot of things that are involved [rape]*! *There are hits*, *blows*, *head-butts*, *and the whole of these to cause her to open her legs (laughter and whispering)… So you can kill her*.*” (Male*, *54*, *Electrical Engineer*, *non-IDP)*

*“Because for someone to force a women to have sex with her*, *he will have to brutalize her*. *He will not be gentle*, *he will brutalize her and the women will suffer pains… he will throw your legs apart… it’s tiresome and you will necessarily be affected*. *You will not be able to do anything in the morning*.*” (Female*, *42*, *Housewife*, *non-IDP)*

Focus group participants provided additional examples of the interplay between different kinds of violence within intimate partner relationships. For example economic IPV often led to physical IPV, resulting in deleterious physical and emotional health impacts. In post-crisis Côte d’Ivoire, economic opportunities are scarce and in some cases, women have become the financial providers for their families [see Table 1]. While women welcomed opportunities to have more control over resources in their relationships, they also described being perceived as a threat by their partners. Among men, this perceived loss of control and traditional gender responsibilities was discussed as an underlying cause of all forms of IPV.

#### IPV and everyday activities

In addition to IPV having specific physical and mental health repercussions for women, IPV also impacted their ability to perform everyday activities including household chores, work, and family tasks. Similar to perceptions of how different forms of IPV impacted health; physical and sexual forms of IPV were viewed as being more disruptive to the completion of everyday activities when compared to economic and emotional IPV. When asked to compare economic IPV and emotional IPV, four of the five female focus groups identified emotional IPV as more disruptive to everyday activities. In contrast, in the same comparison, four of five of the men’s focus groups identified economic IPV as more disruptive for women, suggesting that women and men view the repercussions of emotional and economic IPV differently. As with shame and health, participants described situations in which all forms of IPV impacted the daily activities and obligations of women. In the quote below, a woman describes how emotional IPV can disrupt daily activities.

*“Insults hurt too*. *They really hurt*. *The man will insult you in such a way*, *with wrong words*. *You are obliged to keep them to yourself*, *you can’t tell them to anybody*. *And you keep on thinking about those words*. *Often you start to attend to your activities but when you remember the whole words he used to called you*, *you no longer have the strength to continue*…*you get sad*, *you're disheartened*…*you no longer have the strength to continue*. *You wonder why your husband too could use such ugly words against you*. *This is not good*. *It hurts and it prevents you from doing your activities*.*” (Female*, *38*, *Dyer*, *Non-IDP)*

Economic IPV, most commonly in the form of partners taking money from their wives, limited women from completing daily tasks by preventing women from purchasing items, or leave the house.

*“Snatching money [economic IPV]*, *because without money*, *I cannot do business*, *while if he insults me*, *this won’t have any effect on my work” (Female*, *39*, *Trader*, *IDP)*

All five of the women’s focus groups thought that physical IPV was more disruptive than economic IPV. However, two of the men’s focus groups were unable to reach a consensus regarding the most disruptive form of IPV, with three groups identifying physical IPV as more disruptive to daily activities than economic IPV).

*“If he forces you to have sex with him*, *you will be too exhausted to do anything else*. *If I am forced*, *I do not move*, *I will stay at home*, *because I will be tired*.*” (Female*, *54*, *Trader*, *non-IDP)*

## Discussion

Approximately one third of women in this study reported past year physical and/or sexual IPV, and slightly more than half reported physical, sexual, and/or emotional IPV; findings that are consistent with the global prevalence of IPV [20]. Focus group discussions revealed that physical, sexual, and emotional IPV, in addition to less recognized forms of violence against women such as economic abuse and discrimination from community and family members, were common problems faced by crisis-affected women. Shame and social stigma played an important role in how IPV was perceived and experienced. For women in this study shame and social stigma were often the most important factors for women when discussing the most harmful types of IPV. Public abuse was almost universally viewed as more harmful to health, more shameful, and more disruptive to everyday activities than non-public IPV.

In previous qualitative research with rural men in Côte d’Ivoire, men reported that the erosion of traditional gender norms in conjunction with financial stress and armed conflict were a challenge to their sense of masculinity [21]. In a recent multi-country study among displaced populations, displacement was believed to increase IPV because it resulted in the removal of men from their traditional role as heads of household and financial providers, resulting in more women contributing to the financial well-being of their families. When women gained financial and household responsibility and some degree of empowerment, increased tensions were observed between partners, in turn increasing the risk of IPV [8]. Within this previous study, women’s perceptions of how changing social and gender roles influenced IPV post-crisis were mixed, as some women believed that empowerment and increased independence as a result of the crisis reduced IPV perpetration, while others cited changing gender roles and responsibilities post-crisis as risk factor for increased IPV [7]. However, in the present study, changing gender roles were most commonly discussed as catalyzing IPV, and not as protective against violence or abuse from partners.

Although sexual violence in conflict settings often receives the focus of organizations, media and donors, we found that IPV resulting in shame or stigma, particularly emotional IPV, was also frequently cited concern for crisis-affected women [3]. Shame and stigma were common themes in all of the focus groups and IPV perpetrated in public or that was public knowledge, was perceived as shameful, regardless of the type. Conflict affected women in focus groups conducted in Democratic Republic of the Congo described a similar phenomenon [22]. The women were specifically asked about experiences and repercussions related to sexual violence during the Congolese war, and they reported that community gossip and ridicule was particularly hurtful. In their case, shame as a result of the sexual violence was of equal concern as the attack itself [22].

Further, while not always explicitly discussed in the focus groups, it’s important to consider the broader structural inequalities women regularly that can influence and perpetuate their experiences of IPV [1]. As is seen in Abidjan, social norms can also play a role. Women in the focus groups who had experienced IPV found little support in the surrounding community–yet instead were blamed for the violence perpetrated against them. This sort of public shaming reinforces IPV as a legitimate means for control, limits women’s ability to seek help, and reinforces women’s perceived and actual lower social status. It is only with this lens in mind that we can truly understand the nature of IPV in Abidjan and identify the most effective way to prevent it.

Study results have important implications for practitioners and researchers. For practitioners, it is critical to recognize the interplay between different kinds of violence within intimate partner relationships, as results indicate that it is likely that a woman may be experiencing multiple forms of IPV at once. Furthermore, economic and emotional IPV may be less visible than physical IPV. Response services and interventions need to work holistically around women’s needs and experiences and be designed to address all aspects of violence. As noted in the focus groups, men and women may have different perceptions of IPV consequences and seriousness. Thus, these discrepancies should be addressed within programming that seeks to engage men and women separately.

Programmatic interventions should also take into account the barriers and consequences of disclosure for IPV survivors, including the role of stigma and shame in the decision to disclose IPV experiences. Attention should be paid to creating an environment where women feel safe and supported to disclose, and to strengthening relationships between women to increase peer support, with a focus on confidentiality and solidarity. It is important to recognize other ‘less serious’ forms of IPV and make sure that both women feel comfortable disclosing experiences without jeopardizing their safety.

This study utilized both free listing and pair-wise ranking which allowed participants to identify and prioritize the issues that were most relevant to them. Future GBV research may consider utilizing these qualitative methods which allowed for the facilitation of a structured conversation about sensitive topics and provided insight into the perception of frequency, severity, and relevance of IPV, discrimination, and other problems faced by crisis-affected women. While research regarding sexual and physical violence exacerbated by crisis situations is still necessary, future studies should also include the investigation of additional forms of IPV and discrimination given their relationship to poor health outcomes and disruption of daily activities.

Limitations must be acknowledged. The samples for the quantitative survey and focus groups were small and not representative. Therefore, findings cannot be generalized. Two researchers performed qualitative analysis independently, however pair-wise ranking results were not further analyzed with quantitative software. As with all research on stigmatized behaviors, IPV experiences may have been under-reported. Finally, this was one of the first times this methodology was used to examine how people perceive different forms of IPV, thus additional research is needed.

In summary, this study highlighted the high frequency of past year IPV and provided important insights into how women and men perceive different forms of IPV in a post-crisis and urban setting. Violence experienced by women included physical, sexual and emotional IPV as well as economic IPV, community discrimination, and arguments with partners. Lack of financial resources and unemployment were common problems among crisis-affected women and described as an underlying source of IPV. This research also highlights that women often experience different forms of IPV concurrently. Finally, both women and men reported that shame and stigma play a large role in how women experience the repercussions of IPV, with public episodes of IPV almost always seen as more detrimental than private episodes of IPV. These results underscore the need for increased social support mechanisms for women to reduce shame, stigma, and isolation associated with their experiences. The creation of safe and supportive spaces for women to talk about and challenge social norms may be an important first step in reducing community shaming and the secrecy that often surrounds IPV. Safe spaces along with broader societal outreach, including challenging men’s social positions and creating opportunities for increasing economic resources can, in turn potentially decrease the frequency of IPV and its deleterious impacts on a woman’s well-being.

## Supporting Information

S1 Survey Intimate Partner Violence Questionnaire, July 2012. (DOCX)
