# Supplementary material for: Perceptions and Experiences of Intimate Partner Violence in Abidjan, Côte d'Ivoire
Source: PLoS One. 2016 Jun 16;11(6):e0157348. doi: 10.1371/journal.pone.0157348 (PMC4911101; doi:10.1371/journal.pone.0157348)
Supplement: S1 Survey — (DOCX) [file pone.0157348.s001.docx]

| **Yale School of Public Health and Innovation for Poverty Action** |
| --- |
| Economic and Health Study en Cote d’Ivoire Rurale  (Sections 3 and 4) |
| Une étude conduite en coopération avec IRC  (International Rescue Committee)- Cote d’Ivoire  This questionnaire contains items (as originally created an/or modified) that were developed by the LHSTM.  Mazeda Hossain, Cathy Zimmerman, Ligia Kiss, Charlotte Watts. Life Experiences in Cote d'Ivoire. London School of Hygiene & Tropical Medicine. London. 2010.  **July 2012**  *July 2012* |

Contents

[QUESTIONNAIRE A: WOMEN WITH A PARTNER 3](#_Toc327809779)

[QUESTIONNAIRE A: FEMMES AYANT UN PARTENAIRE 3](#_Toc327809780)

[ADMINISTRATIF 6](#_Toc327809781)

[Section 1. QUESTIONNAIRE DU MENAGE/ *Household questionnaire* 7](#_Toc327809782)

[SECTION 2. VOUS ET VOTRE PARTENAIRE / You and your Partner 9](#_Toc327809783)

[SECTION 3. EXPERIENCE DE VIOLENCE DANS LE COUPLE / *Violence in the couple* 11](#_Toc327809784)

[SECTION 4. REACTIONS /*Reactions* 14](#_Toc327809785)

[SECTION 5: CLIMAT D’INTIMIDATION DANS LE COUPLE/ Climate of intimidation 18](#_Toc327809786)

[SECTION 6. AMIS ET SUPPORT SOCIAL / *Friends and social support* 21](#_Toc327809787)

[SECTION 7. EXPERIENCE AVEC LES ACTIVITES D’EPARGNE ET DE CREDIT DANS LES GOUPES AVEC/ *Expérience with crédits and share outs in the VSLA groupe* 24](#_Toc327809788)

[SECTION 8. AUTONOMIE FINANCIERE / Financial autonomy 28](#_Toc327809789)

[SECTION 9. INSECURITE ALIMENTAIRE / Food Insecurity 32](#_Toc327809790)

[SECTION 10 : SANTE DE LA REPRODUCTION/ Reproductive Health 37](#_Toc327809791)

[SECTION 11: PLANNING FAMILIAL/ *Family Planning* 38](#_Toc327809792)

[SECTION 12: VIH ET IST/ *HIV and STI’s* 39](#_Toc327809793)

[Section 13. BIEN ETRE EMOTIONNEL / Emotional welfare 44](#_Toc327809794)

[Section 14. AUTRES EXPERIENCES DANS VOTRE VIE/ Other life experiences 46](#_Toc327809795)

[SECTION 15.LES DECISIONS DANS LE MENAGE / Decisions in the household 47](#_Toc327809796)

[SECTION 16. NORMES SOCIALES/ *Social norms* 48](#_Toc327809801)

[Section 17. CONSOMMATION ET BIENS/ *Consumption and Assets* 50](#_Toc327809802)

[Section 18. CLOTURE DE L’ENTREVUE / *End of the interview* 55](#_Toc327809803)

# QUESTIONNAIRE A: WOMEN WITH A PARTNER

# QUESTIONNAIRE A: FEMMES AYANT UN PARTENAIRE

**HEURE DE DEBUT:**

**Starting time**

**FORMULAIRE DE CONSENTEMENT**

Bonjour, je m’appelle.....

- J’aimerais vous inviter à participer à une étude conduit par une université américaine (Ecole de santé publique de Yale)
- Dans cette étude, on veut en savoir plus sur la manière dont les décisions se prennent dans les ménages en Cote d’Ivoire. Cela va nous aider à mieux développer des programmes pour favoriser le développement économique des femmes.
- Vous êtes invites parce que vous faites partie du groupe des femmes crée par IRC.
- Si vous acceptez de participer, un enquêteur qui comprend votre ethnie va discuter avec vous pendant environ une heure.
- On va vous discuter de sujets tels vos dépenses, les relations que vous entretenez avec votre partenaire ou votre famille.
- Comme on va parler de choses personnelles telles que votre argent, la manière dont vous le dépensez, vos relations avec votre mari, certaines questions vont peut être vous mettre mal a l’aise, vous énerver ou vous rendre tristes.
- J’aimerais vous assurer que tout ce nous allons dire restera entre vous et moi. Votre nom n’apparaitra sur aucune des reponses.
- Vous avez le droit d’arrêter la discussion à tout moment, ou me demander de sauter certaines questions auxquelles vous n’aimeriez pas répondre. En plus, vous pouvez aussi faire des pauses à tout moment.
- Si vous arrêtez la discussion ou sautez certaines questions, il’ n’y aura aucune punition pour ca. Ca ne vous empêchera pas de participer au groupe des femmes crées par IRC. En plus, si vous décidez plus tard de ne plus être dans cette étude, vous pouvez aussi appeler notre bureau et on effacera touts les informations que vous nous avez donne
- Il n’y aura pas de compensation directe (comme l’argent par exemple) parce que vous avez participe à cette étude. Néanmoins, votre participation et vos informations permettront d’améliorer les programs existants dans des communautés telles que les vôtres. A la fin de l’interview, on va aussi vous donner une liste de personnes ressources qui peuvent vous offrir les services dont vous aurez besoin.

Questions:

Il ya deux personnes clés que vous pouvez appeler si vous avez des questions à poser a propos de l’enquête. Il ya Mr Patrice Boa et Mme Jhumka Gupta dont les numéros de téléphone et adresse sont sur les formulaires de consentement que je vais vous remettre. On va également vous donner une liste de personnes qui travaillent sur ce projet et qui sont dans cette ville avec vous. Vous pouvez aussi les appeler si vous avez des questions.

(INDIQUEZ A LA PARTICIPANTE OU MARQUER LES CROIX SUR LES FORMULAIRES DE CONSENTEMENT QUE VOUS ALLEZ LEUR REMETTRE)

Si vous acceptez de participer à l’étude, marquez s’il vous plait une croix (X) au niveau de la première déclaration ci-dessous. Ce formulaire de consentement est à garder pour votre future référence.

Si vous décidez de ne pas participer à cette étude, marquez s’il vous plait une croix (X) au niveau de la seconde déclaration ci-dessous. Vous confirmez que l’équipe de recherche vous a explique le projet de recherche. Vous avez lu et compris ce formulaire de consentement. Si a tout moment, vous décidez de ne plus participer à cette étude, vous pourrez retirer ce formulaire de consentement.

| **Avez-vous des questions pour moi maintenant?**  *Do you have any questions for me?* | - Non | - Oui   **CONTINUEZ** |
| --- | --- | --- |
| **Êtes-vous êtes d’accord pour participer à l’entretien?**  *Do you agree to participate in this interview?* | - Non   **FIN – LE REMERCIER POLIMENT** | - Oui |
| **Est-ce que nous pouvons commencer maintenant?**  *May I begin ?* | - Non   **PRENDRE RENDEZ-VOUS** | - Oui |
| **Il est très important de dire la vérité pour toutes les questions que nous allons vous poser ! Acceptez-vous de nous donner des réponses sincères?**  *It is very important that you are honest with your responses. Will you agree to provide honest answers*? | - Non | - Oui |
| **Il est très important qu’on soit vraiment seul pour parler. Est-ce qu’on peut le faire ici?**  *It is very important that we speak in a private place. Can we do that here?* | - Non   **TROUVER UN ENDROIT ISOLE** | - Oui |

**A COMPLETER PAR L’ENQUETEUR**

**Je certifie d’avoir lu la demande d’autorisation ci-dessus et que l’enquêté est d’accord pour participer.**

Date: _________________________

Nom de l’enquêteur: _________________________

Signature de l’enquêteur: _________________________

| SECTION 3. EXPERIENCE DE VIOLENCE DANS LE COUPLE / *Violence in the couple* | | | |
| --- | --- | --- | --- |
| **LISEZ : Dans une relation, on peut avoir des disputes. On peut faire des histoires et même s’insulter ou devenir violent. C’est de ce genre d’expérience qu’on va maintenant parler (les palabres, les histoires dans le couple). Comme je l’ai déjà dit, je tiens encore à te rassurer que tout ce qu’on va dire, toutes les réponses que tu vas me donner vont rester entre nous ; tes réponses vont rester secrètes. Personne ne saura ce qu’on s’est dit, même pas ton mari ou bien un membre de ta famille.**    *READ ALOUD: In every relationship, there are conflicts and arguments. Sometimes, people have very difficult experiences when such conflicts and arguments arise, including insults and violence. The next set of questions will ask you about these kinds of experiences. Remember, all of your responses will be kept private, and will not be shared with your partner or family* | | | |
| **VIOLENCE EMOTIONELLE SUBIE/EMOTIONAL (PSYCHOLOGICAL) VIOLENCE EXPERIENCED** | | | |
|  | **Durant l’année dernière, est ce que ton partenaire actuel ou ton partenaire le plus récent, t’ a fait l’une des choses suivantes ?**  *In the past year, has your current or most recent partner done any of the following to you?* | **OUI/ Yes** | **NON/ No** |
| 301 | **A essayé de t’empêcher de visiter tes ami(e)s?**  *Tried to forbid you from visiting your friends?* | 1 | 0 |
| 302a) | **T’ a effrayé ou intimidé (par exemple, en te regardant d’une certaine manière, en criant ou en cassant quelque chose)?**  *Done something to frighten or intimidate you? (For example, in the way she/he looks at you or by yelling or breaking something?)* | 1 | 0 |
| b) | **A menacé de te blesser ou de blesser quelqu'un qui est proche de toi, quelqu’un qui t’est cher?**  *Threatened to hurt you or someone you care about?* | 1 | 0 |
| c) | **T’ a diminué ou humilié devant d’autres personnes?**  *Belittled or humiliated you in front of other people?* | 1 | 0 |
| 303 | **Résumé Violence émotionnelle : La participante a t-elle subi la violence émotionnelle (elle a répondu oui a au moins 1 des questions précédentes) ?**  *Summary of emotional violence : Did the participant experience past year emotional violence ( she answered “yes” to at least one of the above question)* | 1 | 0 |

| 304 | **VIOLENCE PHYSIQUE SUBIE/ PHYSICAL VIOLENCE EXPERIENCED** | | |
| --- | --- | --- | --- |
|  | **Durant l’année dernière, est ce que ton partenaire actuel ou ton partenaire le plus récent vous a fait l’une des choses suivantes ?**  *In the past year, has your current or most recent partner done any of the following to you?* | **OUI** *Yes* | **NON**  No |
| a) | **Vous a giflé ou jeté quelque chose qui pourrait vous faire mal?**  *Slapped you or thrown something that could hurt you, or hit you with something that could hurt you?* | 1 | 0 |
| b) | **Vous a poussé ou secoue?**  *Pushed ,shoved, kicked, or dragged you?* | 1 | 0 |
| c) | **Vous a étranglé ou a causé des brûlures intentionnellement?**  *Choked you or burned you intentionally?* | 1 | 0 |
| d) | **Vous a menacé d’utiliser un fusil, un couteau, ou une autre arme contre vous?**  *Threatened to use a gun, knife or other weapon against you?* | 1 | 0 |
| e) | **A utilisé un fusil, un couteau, ou une autre arme contre vous?**  *Used a gun, knife or other weapon against you?* | 1 | 0 |
| 305 | **Résumé Violence physique: La participante a-t-elle subi la violence physique (elle a répondu oui a au moins 1 des questions précédentes) ?**  *Summary of physical violence : Did the participant experiencs past year physical violence ( she answered “yes” to at least one of the above question)* | 1.Oui/Yes | 🡪306 |
|  |  | 0.Non/No | 🡪307 |

| 306 | **Durant l’année dernière, est ce que vous avez vécu l’une des choses suivantes a cause de la violence physique exercée par votre partenaire actuel ou votre partenaire le plus récent?**  *In the past year, did you experience any of the following because of physical violence from your current or most recent partner?* | **OUI**  *Yes* | **NON**  *No* |
| --- | --- | --- | --- |
| a) | **Est-ce que vous avez eu des blessures ou des douleurs?**  *Did you have injuries or pain?* | 1 | 0 |
| b) | **Est-ce que vous avez été blesse au visage, à l’ œil, ton pied ou ton bras s’est déplacé? tu a été brulée ? tu as perdu saigne, tu as perdu du sang ou avez-vous perdu du sang?**  *Did you have a black eye, a sprain, a dislocated limb, burns, or did you lose blood?* | 1 | 0 |
| c) | **Est-ce que vous avez eu une blessure profonde, des os cassés, des dents cassés ou d'autres blessures graves?**  *Did you have a deep wound, a broken bone, broken teeth, or other serious injuries?* | 1 | 0 |

| **VIOLENCE SEXUELLE / SEXUAL VIOLENCE** | | | |
| --- | --- | --- | --- |
| **Merci encore de partager toutes ces choses avec moi. Je sais que ce sont des histoires intimes que vous n’êtes pas obligee de partager avec moi. Merci encore pour votre disponibilité. Mais on va encore parler de choses plus intimes, de tes rapports avec ton mari/partenaire. C’est vraiment ta vie intime, c’est pourquoi j’aimerais que tu me donnes la permission de continuer. Est-ce qu’on peut continuer ?**  **I’d like to thank you again for sharing all these experiences with me. I realise these are intimate stories and experiences you are not obliged to share with me. Thanks again for your availability. However, I still need to talk with you about some more intimate experiences with your husband/partner. This is your private life, that’s why I’m asking your allowance for moving forward with the interview. Can we continue?** | | | |
| 307 | **Durant l’année dernière, est ce que votre partenaire actuel ou le partenaire le plus récent vous a fait l’une des choses suivantes ?**  *In the past year, has your current or most recent partner done any of the following to you?* | **OUI**  *Yes* | **NON**  *No* |
|  |  |  |  |
| a) | **Est-ce que vous avez eu des relations sexuelles avec votre partenaire parce que vous vous sentiez menacé ou intimidé par lui?**  *Have you ever been forced to have sex with using threats or intimidation?* | 1 | 0 |
| b) | **Est-ce que votre partenaire vous a forcé de coucher avec lui/elle alors que vous ne le vouliez pas?**  *Physically forced you to have sex when you did not want to?* | 1 | 0 |
|  | **Résumé Violence sexuelle : La participante a-t-elle subi la violence sexuelle ?**  *Summary of sexual violence : Did the participant experience has past year of physical violence* | 1.Oui/Yes |  |
|  |  | 0.Non/No |  |

| BILAN SECTION 3/ SUMMARY SECTION 3 | | | |
| --- | --- | --- | --- |
| 308 | **Résumé SECTION 3 : La participante a-t-elle subi la violence physique et/ou sexuelle:**  *Summary of section 3:Did the participant s experienced past year physical and/or sexual violence.* | 1.Oui/Yes | 🡪Section 4 |
|  |  | 0.Non/No | 🡪Section 5 |

| SECTION 4. REACTIONS /*Reactions* | | | | | | | | | | |
| --- | --- | --- | --- | --- | --- | --- | --- | --- | --- | --- |
| 401 | **Tu m’as dit que l’année dernière, tu as subi la violence physique ou sexuelle de la part de ton partenaire (…rappeler exactement le type de violence tel que décrit a la section 3). J’aimerais savoir si tu en as parle avec quelqu’un ou si tu as demande de l’aide a quelqu’un parce que ton partenaire était violent avec toi.**  **As-tu parle a ou as-tu demande de l’aide a (….lisez la liste ci-dessous et les options) parce que ton partenaire était violent avec toi ?**  **.** | **A) SI OUI: Avec qui vous avez parlé?**  *If yes: who did you talk to?*  **Si O UI, Continuez avec B***.*    **Si NON, passez au point suivant.**  *If He/She answered yes, go to B.*  *If not, go to the following option* | | **B) Cette personne vous a aide?**  *Did this person help you***?**  **Si O UI, Continuez avec C***.*    **Si NON, revenez a A et passez au point suivant.**  *If He/She answered yes, go to C.*  *If not, go back to A and ask the following option* | | **C) Quelle a été la réaction de cette personne?** *What was this person reaction?*  1-**Aucune reaction**/ *No reaction*  2-**Conseille de rester avec mon mari**/*Advised to stay with my husband*  3- **conseille de chercher quelqu’un d’autre pour** **m’aider**/*advised to look for someone else*  4-**m’a propose une place chez lui/elle**/*offert me a place in his/her home*  5-**m’a reconforte**/*was supportive*  6-**a decide de parler a mon partenaire**/*Proposed to talk to my partner*  7-**A decide de demander l’aide d’autres autorites**/ *proposed to look for other authorities to help me*  8-**m’a conseille le divorce**/*Advised me to divorce*  9-**Autre ( a preciser)/***Other*  *( precise)*  10-**Autre ( a preciser**)/ *Other*  *( precise)* | | | | |
|  |  |  |  |  |  |  |  |  |  |  |
|  | *You’ve just told me that you’ve experienced IPV during the last year ( recall exactly what she said at section 3). I’d like to know if you talked to someone about this violence from your partner.*  *did you ask (….read the list and options below) for help because your partner was violent with you* |  |  |  |  |  |  |  |  |  |
|  |  | **Oui** | **Non** | **Oui** | **Non** |  |  |  | **/_______/** | |
|  | **LISEZ LA LISTE ET LES OPTIONS** |  |  |  |  |  |  |  | /_______/ | |
| a. | **Un ou des ami(s) /** *Friend(s) male* | 1 | 0 | 1 | 0 |  |  |  | /_______/ | |
| b. | **Un ou des ami(e)s /** *Friend(s) female* | 1 | 0 | 1 | 0 |  |  |  | /_______/ | |
|  | **Ta mère /** *Mother* | 1 | 0 | 1 | 0 |  |  |  | /_______/ | |
| c. | **Ton père** / Father | 1 | 0 | 1 | 0 |  |  |  | /_______/ | |
| d. | **Ta Sœur /** *Sister* | 1 | 0 | 1 | 0 |  |  |  | /_______/ | |
| e. | **Ton Frère** */ Brother* | 1 | 0 | 1 | 0 |  |  |  | /_______/ | |
| f. | **D’autres hommes de la famille/** *Other males relatives* | 1 | 0 | 1 | 0 |  |  |  | /_______/ | |
| g. | **D’autres femmes de la famille/***Other females relatives* | 1 | 0 | 1 | 0 |  |  |  | /_______/ | |
| h. | **Des Voisin(s)/ N***eighbours* | 1 | 0 | 1 | 0 |  |  |  | /_______/ | |
| i. | **Un Leader religieux/** *Religious leaders* | 1 | 0 | 1 | 0 |  |  |  | /_______/ | |
| j. | **Au Chef du village/** *Chief of village* | 1 | 0 | 1 | 0 |  |  |  | /_______/ | |
| k. | **A un Leader communautaire (i.e., président des femmes, des jeunes, notable)**  *Community leaders ( president of women, youth, villages’ officials)* | 1 | 0 | 1 | 0 |  |  |  | /_______/ | |
| l. | **A un Agent de terrain IRC** *(IRC field staff)* | 1 | 0 | 1 | 0 |  |  |  | /_______/ | |
| m. | **A une autre ONG/ Organisation communautaire ( pas IRC)/** *Other NGO ( No IRC)* | 1 | 0 | 1 | 0 |  |  |  | /_______/ | |
| n. | **Au Médecin/ Infermière/ Sage femme/** *Doctor, Nurse, Midwife)* | 1 | 0 | 1 | 0 |  |  |  | /_______/ | |
| o. | **A un Avocat/** *Lawyer* | 1 | 0 | 1 | 0 |  |  |  | /_______/ | |
| p. | **A un Policier /** *Policeman* | 1 | 0 | 1 | 0 |  |  |  | /_______/ | |
| q. | **A tes enfants/** *My children* | 1 | 0 | 1 | 0 |  |  |  | /_______/ | |
|  | **Autres (préciser):/** *Others, precise*  **[_________________________________________________________]** | 1 | 0 | 1 | 0 |  | | | | |
| X1. | **Autres (préciser):**  **[_________________________________________________________]** | 1 | 0 | 1 | 0 |  | | | | |
|  |  |  |  |  |  |  | | | | |
| 402 | **RESUME DE LA QUESTION 401/ *Summary of the question 401*** | | | | | | | | | |
|  | **La participante a-t-elle parle (ou demande de l’aide) a quelqu’un parce que son partenaire était violent avec elle ?** | **Oui/***Yes* | | | | | | 1 | | **Aller a 404** |
|  | *In the last year, did the participant ask someone for help about the partner violence she was experiencing?* | **Non/***No* | | | | | | 0 | | **Aller a 403** |
|  |  |  |  |  |  |  |  |  |  |  |
|  | **Si tu n’as rien dit a quelqu'un, pourquoi?**  **Investiguez (quoi d’autre ? quoi d’autre ? quoi d’autre ?)**  **ENTOUREZ TOUTES LES OPTIONS MENTIONEES /NE LISEZ PAS LA LISTE. Posez la question et demandez quoi d’autre, quoi d’autre.**  *If you did not tell anything to anyone, why ( give the reasons)*  *Circle all that apply/ Don’t read the list but probe ( what else? What else? What else?)* | | | | | | | | |  |
| 403  A |  |  |  |  |  |  |  |  |  |  |
|  | **J'AI EU PEUR D'ETRE RENVOYE** /*I was afraid he leaves me* | | | | | | | 1 0 | |  |
| B | **MA FAMILLE N'ALLAIT PAS AIMER CELA/***My family would not liked it* | | | | | | | 1 0 | |  |
| C | **JE NE SAVAIS PAS A QUI PARLER/I** *did not know who to talk to* | | | | | | | 1 0 | |  |
| D | **J`AI EU PEUR DES REPRESAILLES/***I was afraid of retaliation* | | | | | | | 1 0 | |  |
| E    F  G      H  I | **JE PENSAIS QUE C`ETAIT MA FAUTE/** *I thought It was my fault* | | | | | | | 1 0 | |  |
|  | **JE NE VOULAIS PAS L'HUMILIER/** *I did not want to humiliate him* | | | | | | | 1 0 | |  |
|  | **LES AUTRES ALLAIENT PENSER QUE C'ETAIT MA FAUTE/** *Others would think that it was my fault* | | | | | | | 1 0 | |  |
|  | **J'AI EU HONTE/** *I was ashamed* | | | | | | | 1 0 | |  |
|  | **J'AI EU PITIE/** *I was merciful* | | | | | | | 1 0 | |  |
| J | **AUTRE (préciser)/Other (precise):** | **[_______________________________]** | | | | | | 1 0 | |  |
| K |  | **[_______________________________]** | | | | | | 1 0 | |  |
|  | **AUTRE (préciser):** |  |  |  |  |  |  |  |  |  |
|  |  |  |  |  |  |  |  |  |  |  |
|  |  |  |  |  |  |  |  |  | |  |

| 404 | **Tu m’as dit que l’année dernière, tu as subi la violence physique ou sexuelle de la part de ton partenaire (rappeler exactement ce qu’elle a dit a la section 3).**  **As-tu utilise ou demande (….lisez la liste ci-dessous et les options) parce que ton partenaire était violent avec toi ?**  *You’ve just told me that you’ve experienced IPV during the last year (recall exactly what she said at section 3). I’d like to know if you used any of the following services because of your partner’s violence*  *In the last year, did you ask/ use (….read the list and options) because of your partner’s violence you were experiencing?* |  | | | **B) Ces services vous ont aidé?** | | |
| --- | --- | --- | --- | --- | --- | --- | --- |
|  |  | *Si OUI, Continuez avec B.*  *Si NON, passez au point suivant.*  *If She answered yes to A., go to B. If not, go to the following option* | | | Did those services help you? | | |
|  |  | **OUI** | **NON** | | **OUI** | **NON** | |
|  | **LISEZ LA LISTE ET LES OPTIONS / Read the list and options** |  |  | |  |  | |
| **A** | **L’aide psychosociale (Discuter avec les gens de COMISSOU)**/ *Counseling (l’écoute)* | 1 | 0 | | 1 | 0 | |
| **B** | **Services médicaux (Aller a l’hôpital)***/ Medical services* | 1 | 0 | | 1 | 0 | |
| **C** | **La police** */ Police* | 1 | 0 | | 1 | 0 | |
| **D** | **Le centre social/** *Social center* | 1 | 0 | | 1 | 0 | |
| **E** | **La justice** */ Legal aid* | 1 | 0 | | 1 | 0 | |
| **F** | **L’aide financière (Est ce que tu as du demander de l’argent a quelqu’un) / Financial help** | 1 | 0 | | 1 | 0 | |
|  |  |  |  | |  |  | |
| **X1** | **AUTRE (préciser)** *Other (precise***):/:**  **[_______________________________]** | 1 | 0 | | 1 | 0 | |
| **X2** | **AUTRE (préciser):**  **[_______________________________]** | 1 | 0 | | 1 | 0 | |
| 405 | **RESUME DE LA QUESTION 404** | | | | | | |
|  | **Au cours de l'année dernière, la participante a utilisé des services après avoir subi la violence exercée sur elle par son partenaire**  **(**In the last year, the participant used some services because of her partner’s violence? | **Ou**i/Yes | | 1 | | Sect. 5 | |
|  |  | **Non**/No | | 0 | | Aller a 406 | |
|  |  |  | |  | |  | |
| 406 | **Si vous n'avez pas utilisé les services après avoir subi la violence, pourquoi?**  **Investiguez (Quelles sont les autres raisons pour lesquelles vous n’avez pas utilise les services ? quoi d’autre ?quoi d’autres ENTOUREZ TOUTES LES OPTIONS MENTIONNEE?)**  *If you did not use services after experiencing violence, why not?*  *Probe (***what is the other reason why you did not use any services ? what else? What else** *CIRCLE ALL THE OPTIONS MENTIONED* | | | | | |  |
| **A** | **J'AI EU PEUR D'ETRE RENVOYE/** *I was afraid he leaves me* | | | | 1 0 | |  |
| **B** | **J’AI EU HONTE/** *I was ashamed* | | | | 1 0 | |  |
| **C** | **MA FAMILLE N'ALLAIT PAS AIMER CELA/** *My family would not like it* | | | | 1 0 | |  |
| **D** | **JE NE SAVAIS PAS COMMENT LES TROUVER/** *I did not know how to find them* | | | | 1 0 | |  |
| **E** | **JE NE SAVAIS PAS QU’ILS EXISTAIENT/** *I did not know they exist* | | | | 1 0 | |  |
| **F** | **C’ETAIT TROP LOIN /** *It was too far* | | | | 1 0 | |  |
| **G** | **ILS N’ETAIENT PAS DISPONIBLE/** *They were not available* | | | | 1 0 | |  |
| **H** |  | | | | 1 0 | |  |
|  | **ILS N’ETAIENT PAS UTILES /***They were not useful* | | | |  | |  |
| **I** | **AUTRE (préciser)/***Other (précise)***: [_______________***______________]* | | | | 1 0 | |  |
| **J** | **AUTRE (préciser): [_______________________________]** | | | | 1 0 | |  |
